# Intranasal Insulin Eases Autism in Rats via GDF-15 and Anti-Inflammatory Pathways

**DOI:** 10.3390/cimb46090624

**Published:** 2024-09-20

**Authors:** Duygu Burcu Arda, Kerem Can Tunç, Mehmet Fatih Bozkurt, Ejder Saylav Bora, Ayşe Çiğel, Oytun Erbaş

**Affiliations:** 1 Department of Pediatrics, Taksim Research and Training Hospital, Istanbul 34365, Türkiye; dr.duyguburcu@hotmail.com; 2 Department of Biology, Faculty of Science, Adnan Menderes University, Aydın 09010, Türkiye; keremtunc3323@gmail.com; 3 Department of Pathology, Faculty of Veterinary Medicine, Afyon Kocatepe University, Afyon 03100, Türkiye; faith.bozkurt@gmail.com; 4 Department of Emergency Medicine, Faculty of Medicine, Izmir Katip Çelebi University, Izmir 35150, Türkiye; 5 Department of Physiology, Faculty of Medicine, Izmir Democracy University, Izmir 35150, Türkiye; aysecigel@gmail.com; 6 Department of Physiology, Faculty of Medicine, Demiroğlu Bilim University, Istanbul 34381, Türkiye; oytunerbas2012@gmail.com

**Keywords:** autism, hippocampus, intranasal insulin, propionic acid

## Abstract

In rat models, it is well-documented that chronic administration of propionic acid (PPA) leads to autism-like behaviors. Although the intranasal (IN) insulin approach is predominantly recognized for its effects on food restriction, it has also been shown to enhance cognitive memory by influencing various proteins, modulating anti-inflammatory pathways in the brain, and reducing signaling molecules such as interleukins. This study seeks to explore the potential therapeutic benefits of IN insulin in a rat model of autism induced by PPA. Thirty male Wistar albino rats were categorized into three cohorts: the control group, the PPA-induced autism (250 mg/kg/day intraperitoneal PPA dosage for five days) group, treated with saline via IN, and the PPA-induced autism group, treated with 25 U/kg/day (250 µL/kg/day) insulin via IN. All treatments were administered for 15 days. After behavioral testing, all animals were euthanized, and brain tissue and blood samples were collected for histopathological and biochemical assessments. Following insulin administration, a substantial reduction in autism symptoms was observed in all three social behavior tests conducted on the rats. Moreover, insulin exhibited noteworthy capabilities in decreasing brain MDA, IL-2, IL-17, and TNF-α levels within autism models. Additionally, there is a notable elevation in the brain nerve growth factor level (*p* < 0.05) and GDF-15 (*p* < 0.05). The assessment of cell counts within the hippocampal region and cerebellum revealed that insulin displayed effects in decreasing glial cells and inducing a significant augmentation in cell types such as the Purkinje and Pyramidal cells. The administration of insulin via IN exhibits alleviating effects on autism-like behavioral, biochemical, and histopathological alterations induced by PPA in rats. Insulin-dependent protective effects show anti-inflammatory, anti-oxidative, and neuroprotective roles of insulin admitted nasally.

## 1. Introduction

Autism Spectrum Disorder (ASD) is a collection of inherited neurobehavioral diseases characterized by challenges in social communication, as well as the prevalence of repetitive behaviors and inflexible patterns [1]. Historically, ASD has been regarded as a condition that impairs functioning. The current classification of autism spectrum disorder (ASD) in the DSM-5 criteria (APA, 2013) consists of three severity levels: needing support, requiring significant assistance, and requiring very substantial support [2]. While the Centers for Disease Control and Prevention (CDC) estimated the prevalence of ASD at 0.6% in 2000, by 2018, this estimate had increased to 2.3%, making it one of the most prevalent neurodevelopmental disorders among children in the United States, surpassing intellectual disability [3]. This condition is typically diagnosed in early childhood and is genetically diverse, with a prevalence in males that is four times higher than in females. Autism has a complex etiology, with genetics playing a significant role; heritability could be as high as 90%, and numerous genes have been linked to this disorder [4]. This disorder results from complex interactions between genetic and environmental factors, including epigenetic influences. Advancements in genetic technology and testing have facilitated the identification of specific underlying causes in approximately 40% of individuals with ASD who seek genetic evaluation [4]. This progress has been achieved through a comprehensive three-tiered clinical genetics approach. Both metabolic disorders and molecular or cytogenetic abnormalities frequently offer valuable insights into the etiology of ASD [5].

To understand the etiology of neurological disorders like ASD and to develop effective treatments, experimental models using rats are commonly employed to investigate the mechanical processes involved. Among these models, propionic acid (PPA) is notable [6]. PPA, produced by gut bacteria, can traverse the gut-blood and blood-brain boundaries. Prior studies have shown that when adult rats are repeatedly given PPA, it leads to behavioral and neuropathological alterations that resemble those observed in individuals with ASD-like symptoms. Autism Spectrum Disorder is used only as a model in rats, and ASD-like behaviors are observed in rats following PPA administration. These alterations include increased activity levels, stereotypical behavior, and repetitive activities [7,8]. The mechanisms by which PPA induces these changes include its ability to enter the central nervous system (CNS) via monocarboxylate transporters, inhibit the Na+/K+ ATPase, enhance the N-methyl-D-aspartate receptor sensitivity, alter mitochondrial and fatty acid metabolism, and affect gene expression.

Furthermore, PPA accumulation in cells can lead to intracellular acidification, potentially impacting neurotransmitter release, disrupting neural synaptic connections, and increasing intracellular calcium release [6]. These factors can collectively influence neuronal communication and behavior, contributing to various neurodegenerative disorders. Consequently, PPA is recognized as a significant component in developing experimental animal models for ASD [9,10].

While behavioral therapies remain the primary approach for managing ASD, there is a clear need for pharmacological treatments. Psychotropic medications are used to address symptoms such as aggression, repetitive behaviors, low frustration tolerance, hyperactivity, and inattention [11]. However, specific drugs or psychosocial interventions directly targeting the core symptoms of ASD are still lacking.

Insulin affects the brain, heart, kidneys, bones, skin, and hair follicles and regulates blood sugar and diabetes [12,13,14]. It was initially thought to be produced solely by pancreatic β cells. Insulin is found in small amounts in CNS neurons, allowing it to control the CNS and have pro- and anti-atherogenic effects on the vascular system [13,15,16]. According to recent studies, this hormone affects glucose absorption in the spinal cord, pituitary, and pineal glands. Intranasal (IN) CNS insulin administration reduces insulin absorption and environmental impact. Insulin delivered intranasally accumulates rapidly in the cerebrospinal fluid via the olfactory and surrounding routes, suggesting brain delivery [17]. Intranasal insulin reduces food intake, body weight, and glucose balance in healthy people and patients [17]. Insulin controls synaptic activity in the hippocampus, cerebellum, and prefrontal cortex across the blood-brain barrier [18,19]. These properties of insulin improve memory in healthy people and those with moderate cognitive impairment or Alzheimer’s disease [20]. Rapid, repetitive insulin administration appears safe. Insulin affects sleep, sensory perception, mood, and neuroendocrine function [20]. Directly injecting insulin into the brain’s ventricles reduces liver glucose production [21]. Insulin affects the central nervous system’s gluconeogenesis enzymes. In another experiment, 160 IU of insulin reduced food consumption in healthy men but not women [22]. Women remember better than men. A concentrated insulin dose may affect central nervous system energy and memory, with gender differences.

Research shows that obese men and women have similar cerebral insulin transport [22]. The study examined how IN insulin affects PMS, which has symptoms like autism spectrum disorder. Children three and older showed cognitive and social improvements after six months of insulin therapy [23,24]. The cognitive enhancement provided by insulin in both healthy and diseased individuals, as well as its various positive effects on conditions such as dementia and Alzheimer’s disease, is related to its direct impact on the cerebellar and hippocampal regions of the brain through IN administration [20]. For instance, in ASD, pathways such as AKT-mTor, subject to hyperactivation, show reduced neuroinflammation when interacting with molecules like the insulin-like growth factor-1 (IGF-1) [25]. This process is mediated by changes in cytokines such as IL-6, IL-17, and IL-2, as well as alterations in microglial function. The IGF-1 has begun to demonstrate its effectiveness in alleviating symptoms also in children with Rett syndrome, a genetic condition that has several clinical similarities to ASD [25].

Furthermore, the IGF-1 (1–3) compound is undergoing controlled clinical trials in the United States. The United States Food and Drug Administration (FDA) has awarded Fast Track and Orphan Drug designation to the IGF-1 for treating Rett syndrome, also known as (RS) and Fragile X syndrome. The European Commission has officially granted Orphan Drug designation to the IGF-1 (1–3) for treating RS and Fragile X syndrome in the European Union. According to sources, no safety issues have been reported [26,27].

Based on this information, the present study examines the impact of directly administering insulin through the intranasal route on ASD in a rat experimental model. The hypothesis of the present study is that insulin, when administered directly to the brain, can be used as a therapeutic approach in neurodevelopmental disorders such as ASD. This study is the first and only research that quantitatively assesses the physiological impacts of intranasal insulin administration in a rat model of autism induced by PPA. This study covers various parameters, including biochemical, histopathological, and behavioral tests.

## 2. Materials and Methods

### 2.1. Animals

This study employed 30 male 150–200 g Wistar albino rats aged 10–12 weeks. It followed the National Institutes of Health’s Guidelines for the Care and Utilization of Laboratory Animals. With the approval of the Science University’s Animal Ethics Committee (3023014902), the Experiment Animal Laboratory at the Science University provided the rats. The rats were kept in steel cages with controlled temperatures (22 ± 2 °C) and 12-h light/dark cycles, fed ad libitum.

### 2.2. Experimental Procedures

Studies have shown that autism is more frequently observed in males; therefore, to better simulate the condition, only 30 male Wistar albino rats were used in the present study. Rats received 250 mg/kg/day intraperitoneally of propionic acid to produce autism for five days. Twenty rats received PPA to develop an autism model. Ten rats were normal controls and had no surgery. Rats administered PPA were randomly split into two groups. The study groups were designed as follows: Group 1: Oral control (n = 10); Group 2 (PPA + saline, n = 10): PPA + 250 µL/kg/day 0.9% NaCl saline intranasally; Group 3 (PPA + Insulin, n = 10): PPA + 25 U/kg/day [28,29]. Regular intranasal insulin (100 U/mL, Lilly Humulin R Indianapolis, Indiana, USA). All treatments lasted 15 days. After 15 days, the conduct was tested. All behavioral trials were performed between 10 AM and 3 PM.

After the research, all animals were sacrificed (cervical dislocation) with anesthesia of (100 mg/kg, Ketasol, Richterpharma AG Austria)/xylazine (50 mg/kg, Rompun, Bayer, Germany), and samples of their blood were obtained by heart puncture for biochemical examination.

### 2.3. Behavioral Tests

#### 2.3.1. Three-Chamber Sociability Test

**Figure 1:**
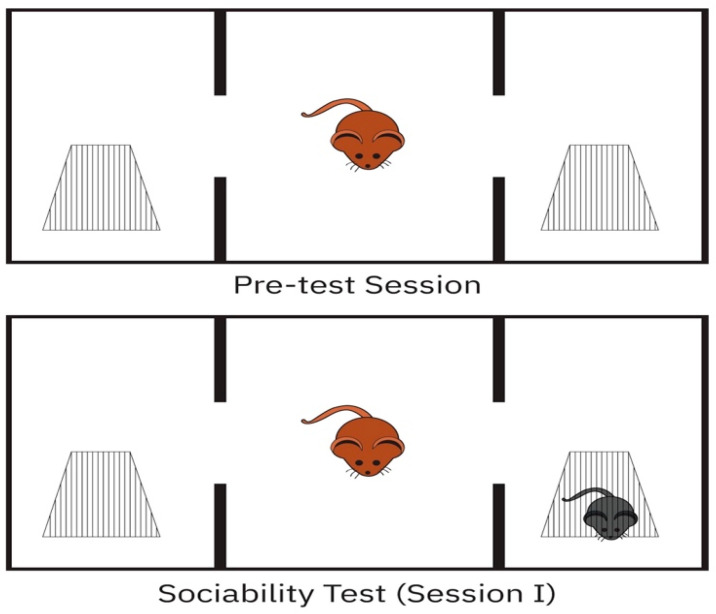
Sociability test.

Sociability was tested as previously reported, with minimal adjustments [30]. A Plexiglas cage (40 cm × 90 cm × 40 cm) was split into three equal parts. Pre-test rats were habituated in the test cage for 5 min on day 1. After 24 h, a stranger rat (Stranger 1) was placed in a tiny plastic cage with mesh-like apertures in one side compartment and an empty cage in the third room to evaluate friendliness. Then, the experiment rat was put in the central chamber and monitored for 10 min in each location (Session I). The test rat was in after entering the chamber with the head and two hind paws. Between tests, the field floor was wiped with 70% alcohol and dried with a paper towel to eliminate olfactory cues from the preceding rat (Figure 1)—stranger time %.

#### 2.3.2. Open-Field

Popular behavioral tests for locomotion and exploration include the open-field (OF) paradigm. Observing altered OF behavior is easy, but determining the causes is difficult. The first is a positive exploratory drive from rodents’ natural desire to explore new environments (for food and shelter), and the second is the animal’s natural tendency to avoid open and brightly lit spaces (exposure to predators). In the autism model, the open-field test can identify motor stereotyped behavior, recurrent auto grooming, and research limitations [30]. A 50 × 50 × 40 cm open-air box was used for the open-field test. The test began with rats being gently put in the center of the box and allowed to roam the arena for 5 min freely. Then, each rat was monitored for 5 min to assess spontaneous activity. The total ambulation (floor divisions traversed with four paws) was recorded. To prevent olfactory signals, the field floor was wiped between animals with 70% alcohol water and dried with a paper towel.

#### 2.3.3. Passive Avoidance Learning (PAL)

Offspring learning and memory were assessed using the FCA’s passive avoidance learning (PAL) test [31]. The PAL uses fear-motivated avoidance tasks to teach rats not to enter a door that appears secure but leads to a dark chamber with an electric grid system that shocks them. The 20 cm × 20 cm × 20 cm PAL box included dark and lit chambers. Rats usually favored the gloomy chamber over the illuminated section. The guillotine door was opened after a 10-s habituation in the light compartment. The light-dark door was closed when a rat entered. After a 3-s 1.5 mA electric shock, the rat was taken from the dark chamber and returned to its cage. Twenty-four hours later, the rats returned to the PAL box. The rat’s delay from light to dark was monitored, but no shock was given. Up to 300 s were recorded for latency. The rat’s memory was measured by how long it took to avoid the dark chamber.

### 2.4. Hippocampus and Cerebellum Histopathology 

Hippocampus injury was investigated in the hippocampus and cerebellum’s Cornu Ammonis (CA) 1 and CA 3 regions. After behavioral testing, animals were killed, and their brains were preserved for three days in 10% formaldehyde in 0.1 M PBS. They were then placed in 30% sucrose at 4 °C till infiltration. Brains were coronally sliced at 40 μm on a sliding microtome and placed on gelatinized glass slides. Brain slices were treated with H_2_O_2_ (10%) for 30 min to remove endogenous peroxidase activity and blocked with 10% normal goat serum (Invitrogen, Thermofisher Waltham, MA, USA) for one hour at room temperature for GFAP immunohistochemistry. After that, slices were incubated with anti-GFAP (Abcam, Inc., MA, US; 1/1000) at 4 °C for 24 h. The Histostain-Plus Bulk kit (Invitrogen) detected rabbit IgG antibodies, and 3,3′ diaminobenzidine (DAB) visualized the result. All sections were cleaned in PBS and shot with an Olympus C5050 digital camera on a BX51 microscope.

To determine the GFAP immunostaining index, GFAP-positive cells were counted in randomized sections (3–4) of each rat at 40× magnification. One investigator blinded to research groups performed all histopathology exams. An image analysis system (Image-Pro Express 1.4.5, Media Cybernetics, Inc., Rockville, MD, USA) was used in four parts in each group.

Using cresyl violet labeling, an image analysis system counted surviving neurons in six slices per group.

### 2.5. Brain Biochemical Analysis 

The brains were promptly extracted and stored at −20 °C until they could be subjected to biochemical analysis after decapitation. The whole brain tissues were pulverized using a glass homogenizer in 5 times the volume of phosphate-buffered saline (pH, 7.4) and then subjected to centrifugation at 5000× *g* for 15 min to prepare the tissue for analysis. The liquid portion was collected, and the protein content of the brain mixture was measured using bovine serum albumin, following Bradford’s method [31].

### 2.6. Enzyme-Linked Immunosorbent Assay (ELISA)

Commercial rat ELISA kits (Thermofisher, Waltham, MA, USA) were used to measure the amounts of TNF-α, NGF, IL-17, IL-2, and GDF-15 in brain supernatants.

An initial addition of 100 µL of supernatant samples and standards (0, 15.6, 31.3, 62.5, 125, 250, 500, and 1000 pg/mL) to microplate wells coated beforehand was followed by a 120-minute incubation period at room temperature. Following five washes of the plate, we introduced 100 µL of antibody reagent and let it incubate for 30 min at ambient temperature. Then, 100 microliters of streptavidin-HRP conjugate were introduced into each well and left to incubate for 30 min at ambient temperature. Furthermore, we introduced 100 µL of tetramethylbenzidine (TMB) for 15 min, followed by 100 µL of stop solution. A total of 2 mL of sample volume was obtained. Absorbance was measured using the multiscanGo microplate reader manufactured by Thermo Fisher Scientific Laboratory Equipment in New Hampshire, United States, with a wavelength of 450 nm.

### 2.7. Measurement of Brain Lipid Peroxidation (MDA) 

MDA (Abcam, Inc., Waltham, MA, USA) levels as thiobarbituric acid reactive substances (TBARS) were used to quantify brain tissue lipid peroxidation. In summary, trichloroacetic acid and TBARS reagent were introduced to the brain tissue samples, followed by thorough mixing and incubation at 100 °C for 60 min. Following a chilling process on ice, the samples underwent centrifugation at 3000 revolutions per minute for 20 min. The absorbance of the liquid remaining after centrifugation, known as the supernatant, was measured at a wavelength of 535 nanometers. The MDA levels were determined using the standard calibration curve with tetraethoxypropane and expressed as nmol per gram of protein.

### 2.8. Measurement of Brain Protein Levels

Total protein concentration in brain samples was determined according to Bradford’s method, using bovine serum albumin as standard.

### 2.9. Statistical Analysis

The statistical analyses were performed using SPSS for Windows, version 15.0 (SPSS Inc., Chicago, IL, USA). The Shapiro–Wilk’s W test was employed to confirm the normal distribution of the data, and Levene’s test was utilized to verify the homogeneity of variances. The results were displayed as the mean plus the standard error of the mean (SEM). Differences among the groups were evaluated using a one-way ANOVA, followed by a Tukey post hoc test to identify specific group comparisons when significant effects were detected. A *p*-value of less than 0.05 was considered indicative of statistical significance.

## 3. Results

### 3.1. Biochemical Investigations of Brain Tissue

Brain MDA levels were more significant in group 2 (*p* < 0.001) compared to the control and group 3 (*p* < 0.001) and also higher in group 3 than in the control group. Higher brain TNF-α levels were observed in groups 2 (*p* < 0.001) and 3 (*p* < 0.001) compared to the untreated group. Brain IL-2 levels were more significant in group 2 (*p* < 0.001) compared to the control and group 3 (*p* < 0.001). Brain IL-17 levels were greater in group 2 (*p* < 0.01) compared to the control and group 3 (*p* < 0.05). Brain GDF-15 levels were increased in group 3 compared to group 2 (*p* < 0.001) and the control group. The control and group 3 had increased brain NGF levels (*p* < 0.05) compared to group 2 (*p* < 0.001) (Table 1).

### 3.2. Behavioural Tests

The mean sociability test score, measured as the percentage of time spent with a stranger rat, was significantly greater in group 3 (*p* < 0.001) and the control group, compared to group 2 (*p* < 0.001). The control group had superior results on the sociability test compared to group 3. The average score of ambulation in the open field test was higher in both the control group and group 3 (*p* < 0.05) compared to group 2 (*p* < 0.001). The average scores for the Passive Avoidance Learning (PAL) latency were significantly more significant in the control group and group 3 (*p* < 0.001) compared to group 2 (*p* < 0.001). Table 2 demonstrates that the control groups had the most significant ratios in all three tests. In contrast, the rats that received IN insulin had values like those of the control groups (Table 2).

As Tnf 2-17, MDA oxidant stress I increase, social behavior scores worsen. In this study, these parameters were found to be correlated (one-way ANOVA *p* < 0.01).

### 3.3. Neuronal Counts and GLIAL Fibrillary Acidic Proteins

The neuronal count in the CA1 region was significantly greater in the control group and group 3 (*p* < 0.001) compared to group 2 (*p* < 0.001). Additionally, the findings of the control group and group 3 were similar. The number of neurons in the CA3 region was significantly greater in both the control group and group 3 (*p* < 0.05) compared to group 2 (*p* < 0.001). The control group and group 3 had a lower glial fibrillary acidic protein (CA1) immunostaining index than group 2. The difference was statistically significant, with *p*-values of less than 0.05 for the control group and group 3 and less than 0.01 for group 2. The immunostaining index of glial fibrillary acidic protein (CA3) was significantly lower in the control group compared to group 2 (*p* < 0.001) and group 3 (*p* < 0.05). The untreated group and group 3 were in proximity. The Purkinje cell count in the cerebellum was more significant in both the untreated group and group 3, with a statistically significant difference (*p* < 0.05). The Purkinje cell count in the cerebellum was comparable between group 3 and the control group. The glial fibrillary acidic protein immunostaining index in the cerebellum was significantly lower in the control group and group 3 (*p* < 0.05) compared to group 2 (*p* < 0.01). Additionally, the control group and group 3 exhibited similarities. The histological examination findings and additional information are shown in Table 3.

### 3.4. Hippocampus and Cerebellum Histopathology Results

**Figure 2:**
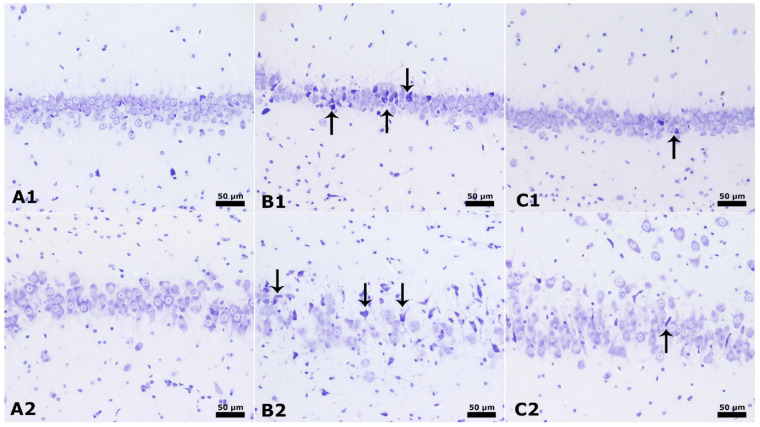
CA1 and CA3 regions of the hippocampus. Cresyl violet stain × 40 magnification. (**A1**,**A2**): Normal control group male rats CA1 and CA3. Normal pyramidal neuron. (**B1**,**B2**): PPA and saline group male rats have neural body degeneration, decreased neural count, and dysmorphological changes in CA1 and CA3 (arrow). (**C1**,**C2**): PPA and Insulin group male rats have increased neural count and improved neural morphology changes in CA1 and CA3 (arrow).

Figure 2 displays clear and identifiable characteristics in the CA1 and CA3 areas of the hippocampus, as shown by the Cresyl violet stain. The rats in the control group displayed conventional pyramidal neurons in the hippocampus. In contrast, the placebo group, which included male rats given PPA and saline injection, showed a significant degeneration of brain cells, decreased neuronal counts, and noticeable structural changes in the CA1 and CA3 areas (as indicated by the arrow in Figure 2). The insulin group, consisting of male rats treated with PPA + insulin, demonstrates increased neurons and improved neuronal morphology in the hippocampus’s CA1 and CA3 regions.

**Figure 3:**
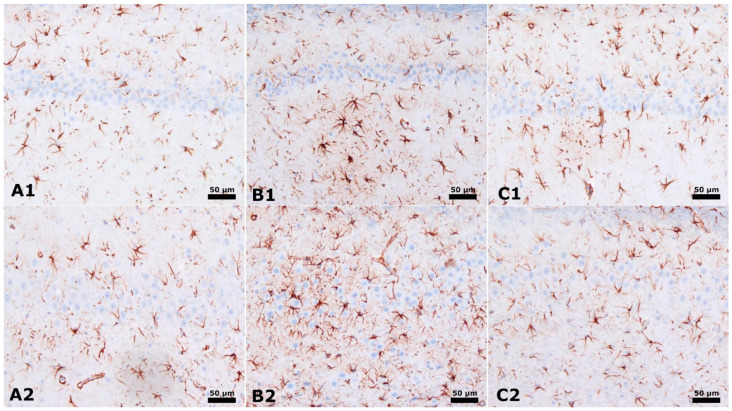
CA1 and CA3 of hippocampus × 40 magnification. Astrogliosis was characterized by GFAP immunostaining (Brown staining). (**A1**,**A2**): Normal control group male rats CA1 and CA3. (**B1**,**B2**): PPA and saline group male rats have increased glial activity in CA1 and CA3. (**C1**,**C2**): PPA and Insulin group male rats have decreased glial activity in CA1 and CA3.

Figure 3 demonstrates the analysis of the CA1 and CA3 areas of the hippocampus, focusing on detecting astrogliosis. This is determined by observing GFAP immunostaining, which is shown by the presence of Brown staining. Within the unaltered group, rats demonstrate conventional glial activity in the hippocampus. On the other hand, in the group of male rats exposed to PPA and saline injection, there is a noticeable increase in glial activity in the CA1 and CA3 areas. The insulin group, consisting of male rats treated with PPA + insulin, exhibits a significant decrease in glial activity in the CA1 and CA3 areas (Figure 3).

**Figure 4:**
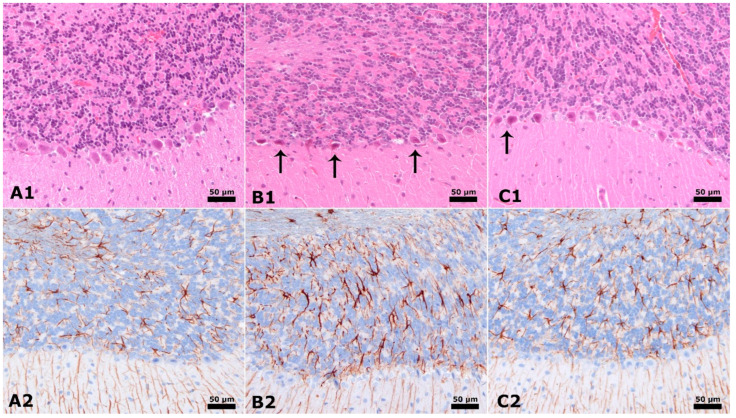
Cerebellum × 40 magnification. Hematoxylin & Eosin and GFAP immunostaining (Brown staining). (**A1**,**A2**): Normal control group male rats’ cerebellum, normal Purkinje neuron. (**B1**,**B2**): PPA and saline group male rats have decreased count, dysmorphological Purkinje Neuron (arrow), and increased glial activity cerebellum. (**C1**,**C2**): PPA and Insulin group male rats have increased count, improved neural morphological changes in Purkinje Neuron, and decreased glial activity in the cerebellum Purkinje Neuron similar to A1-2 (arrow).

Figure 4 illustrates that the cerebellum exhibits different characteristics, as seen by Hematoxylin, Eosin, and GFAP immunostaining. Rats in the control group display conventional Purkinje neurons in the cerebellum. In contrast, the placebo group, which included male rats given PPA and saline injection, exhibited a drop in count, abnormal Purkinje Neuron morphology (shown by an arrow), and increased glial activity in the cerebellum (also indicated by an arrow). The insulin group, consisting of male rats treated with PPA + insulin, exhibits an increased number, enhanced neural structural alterations in Purkinje Neurons, and reduced glial activity in the cerebellum (Figure 4).

## 4. Discussion

In the present study, the potential of IN insulin as a therapeutic agent for ASD was investigated. Although there is currently no definitive pharmacological treatment for ASD, existing therapies are aimed at alleviating symptoms associated with the disorder. It is known that molecules such as insulin and IGF-1 have shown positive cognitive effects in diseases with similarities to ASD. Considering this knowledge, our study is also significant because insulin has not been directly applied to ASD in animal experimental models before, and the application of IN insulin may provide some positive responses in ASD according to the data we have obtained.

In autism animal models, PPA is commonly used as a carboxylic acid. It is generally administered to animals either orally or via intraperitoneal injection. In both our current and some previous studies, autism models have been established using PPA [32,33]. Researchers typically observe the development of the disorder through social and behavioral tests and then evaluate whether the targeted therapeutic agent improves these behaviors using additional social and behavioral tests. It was previously believed that insulin hormone was primarily secreted by pancreatic β cells and that its main role was limited to glucose regulation in diabetes. However, recent studies have challenged this view. Emerging evidence indicates that insulin is also produced by specific neurons in the CNS and plays a role in cognitive functions. A study demonstrated that insulin microinjected into the hippocampus and hypodermic regions of rat groups reduced the Alzheimer-related Aβ1–40 protein expression levels in the CA1 and CA3 regions. Glial activity in CA1 and CA3 decreased, supporting our results [34]. Alzheimer’s disease, multiple sclerosis, dementia, and other neurophysiological illnesses have decreased neuronal activity and increased glial activity. IN insulin crosses the blood-brain barrier. This delivery strategy has been proven to bring ASD-related physiological anomalies closer to average control values.

Tanaka et al. [35] described that effective delivery of oxytocin to the brain by nasal administration is a potential approach for treating autism spectrum conditions. On the other hand, it was found that although the nasal bioavailability of oxytocin is just about 2%, over 95% of oxytocin in the brain is delivered straight from the nasal cavity. Moreover, Yatawara et al. [36] claim that the administration of oxytocin nasal spray greatly enhances social responsiveness in young children diagnosed with autism while causing well-tolerated adverse effects.

TNF-α levels tend to be elevated in neurodevelopmental disorders, indicating an inflammatory response in the CNS. Researchers have observed that in neurodevelopmental disorders such as ASD, TNF-α increases in parallel with certain interleukins. This has led to a search for therapeutics to reduce these elevated parameters [37]. In the present study, it has been demonstrated that IN insulin administration significantly reduces cytokines such as TNF-α, IL-17, and IL-2, which are indicators of inflammation in the brain. Neurotrophic factors like insulin and IGF-1 play a role in early brain development. Furthermore, a study found that IGF-1 levels are lower in children with autism compared to healthy children in a control group [38]. This indicates that molecules such as insulin play a significant role in the pathophysiology of disorders like ASD. In patients with neurodevelopmental disorders such as ASD, the production of insulin in neurons may be more limited compared to that in neurotypical individuals. Therefore, external supplementation with IN insulin might help alleviate ASD symptoms. In another study, the average Homeostasis Model Assessment of Insulin Resistance (HOMA-IR) values in adolescents with ASD were found to be higher compared to those without ASD, regardless of their BMI and pharmacological treatment status [39]. Insulin’s mitogenic properties may contribute to the faster results observed with IN administration. Research conducted on six children diagnosed with 22q13 deletion syndrome found that a one-year therapy with IN insulin significantly improved general and fine motor skills, nonverbal communication, cognitive abilities, and independence. These beneficial effects may also be related to the SHANK3 mutation commonly associated with ASD, as the treatment may address the deficiencies of this mutant gene through inflammatory pathways [39]. The results here provide consistency with the observation that external administration of insulin can reduce autism symptoms. High levels of MDA indicate increased cellular damage and inflammation due to oxidative stress. Elevated MDA levels in brain tissue may be associated with neurodegenerative diseases, mental disorders, and other brain-related conditions and are characterized by elevated values in cases of ASD [40]. As demonstrated in our study, insulin has been shown to reduce MDA levels significantly in autistic rats. Another study assessed oxidative stress among autistic children in Egypt. The research included measuring the amounts of antioxidant enzymes, especially superoxide dismutase (SOD) and glutathione peroxidase (GSH-Px), as well as evaluating lipid peroxidation. The MDA levels were tested for this specific objective. A comparison between a group of 20 children with autism and a group of 25 healthy individuals showed that the levels of SOD and GSH-Px were substantially lower in autistic children compared to the control group. On the other hand, the MDA levels were significantly greater in the autistic children compared to the control group [41]. Consistent with our study, it has been observed that insulin administration reduces MDA levels and normalizes behavior in rats. This effect may be attributed to insulin’s potential role as an antioxidant, which lowers MDA levels, or its ability to increase the levels of SOD and GSH-Px enzymes indirectly. Another commonly observed decrease in neurodevelopmental disorders is the Brain Nerve Growth Factor (NGF) level. Given its crucial role in neuron development, its impairment or reduced expression is characteristic of neurodevelopmental diseases [42]. This reduction helps to explain the loss of neural activity observed in the hippocampus and cerebellum. Insulin administration has been observed to increase Brain-NGF levels to near-normal control values, promote neuronal growth in the hippocampal CA1 and CA3 regions, and repair Purkinje cells in the cerebellum. Additionally, insulin reduces the levels of astrocytes. Insulin receptors are distributed throughout the brain, with the highest concentrations in the olfactory bulb, cerebral cortex, hypothalamus, hippocampus, and cerebellum. These receptor levels decrease with age, suggesting that insulin signaling and IR levels play a role in aging [43]. Studies conducted on rat hippocampal cell cultures have shown that IR signaling promotes cell survival under oxygen and glucose deprivation [44]. Insulin also modifies the levels of postsynaptic α-amino-3-hydroxy-5-methyl-4-isoxazolepropionic acid (AMPA) receptors. Insulin enhances the clathrin-dependent internalization of AMPA receptors, including the glutamate GluR2 subunit, a crucial process for long-term depression mechanisms. This phenomenon has been documented in cultures of hippocampus cells from rats. In addition, infrared (IR) signaling stimulates the incorporation of GABA receptors into the postsynaptic membrane [45,46]. In our research, insulin treatment may have restored damaged neuronal cells and increased the number of neurons by facilitating neurotransmitters like GABA receptors, thereby improving the neural signaling network. This research found that mice receiving intranasal insulin showed enhanced cognitive ability in short-term and long-term item identification tests. Results indicate that delivering insulin directly to the central nervous system (CNS) increases neuronal activity and boosts memory via modifying the levels of KCNA3 [47]. Growth/Differentiation Factor-15 (GDF-15), or macrophage inhibitory cytokine-1, is a distinct member of the growth factor transforming β superfamily. While GDF-15 is typically found in small quantities in most somatic tissues, it is highly expressed in the placenta [48]. GDF-15 expression is often stimulated in response to stress to maintain cellular and tissue balance. Increased concentrations of GDF-15 are often associated with inflammatory and malignant situations, making it a recognized indicator for different pathological states. Consequently, it is a biomarker in certain disorders [48]. The current research revealed a significant rise in GDF-15 levels in rats given insulin. Out of the control group, the PPA group, and the insulin therapy group, the control group had the lowest levels of GDF-15. The GDF-15 levels exhibited a small increase in the rats treated with PPA, but the levels reached their maximum in the autistic model rats that were administered insulin. Research was conducted to assess the quantitative impact of GDF-15 on cognitive functions. Autologous bone marrow mononuclear cells were delivered by intrathecal infusion to evaluate their therapeutic benefits [49,50]. As a result of the application, it was observed that GDF-15 levels decreased, which was not consistent with the findings of our study. Additionally, it is known that GDF-15 has properties related to food restriction [46]. In the current study, the observed increase in GDF-15 following insulin administration may indicate that insulin exerts its effects in the CNS through the modulation of GDF-15. In autism models induced by PPA, GDF-15 levels rise in response to inflammation and oxidative stress in the brain, serving a regulatory function. It is also known that both metformin use and exercise increase GDF-15 levels. Exercise is recognized for its anti-inflammatory effects, and this effect may be mediated through GDF-15. GDF-15 has been shown to signal through the GDNF family receptor α-like (GFRAL) derived from glial cells [48]. The persistence of elevated and even increased GDF-15 levels in ASD rats following treatment may be due to insulin acting as an anti-inflammatory agent via GFRAL. A study investigated GDF-15 levels in patients with posttraumatic osteoarthritis (PTOA). The study demonstrated that antioxidant treatment reduced GDF-15 levels and that p53 activation following oxidative stress led to increased GDF-15 expression. However, external administration of GDF-15 did not produce harmful effects; instead, it induced a pro-regenerative response that enhanced proliferation following cartilage trauma and exhibited protective properties for cartilage and cells [51]. In this context, it has been identified that GDF-15 could be used as a pro-regenerative cytokine in patients with PTOA (p53-induced GDF-15 expression promotes a pro-regenerative response in human chondrocytes upon cartilage injury). In our current study, the increase in GDF-15 levels induced by insulin may have facilitated a pro-regenerative response. Following this initial response, this pathway might have led to increased neuronal levels and the repair of dysfunctional cells. More detailed analyses are required to elucidate the mechanisms of GDF-15 in neurodevelopmental disorders. It is also recognized that GFAP protein expression by astrocytes increases in neurological conditions [52]. Our study found that in rats administered IN insulin, there was a notable decrease in GFAP expression and an increase in the number of neurons, including pyramidal cells in the CA1 and CA3 regions and Purkinje cells in the cerebellum. Other studies have similarly reported increases in these neuronal parameters, consistent with our findings [53,54].

One mechanism of the relation with insulin is described by Stern et al. [55]. An upregulation of the PI3K/Tor pathway, a key intracellular mediator of insulin signaling, is associated with the onset of autism. This route impacts synaptic plasticity and might potentially contribute to ASD in those who are genetically predisposed. Moreover, Manco et al. [39] claim that individuals with ASD have elevated levels of brain insulin resistance, indicating that decreased glucose metabolism in the brain might serve as an indicator of this condition [39]. Zwanenburg et al. [23] found that intranasal insulin significantly impacts social and cognitive skills for kids older than three with Phelan-McDermid syndrome. However, more extensive clinical studies are needed to prove its healing benefit [23]. Similar to these studies, current studies show insulin’s anti-inflammatory, anti-oxidative, and neuroprotective roles, admitted nasally.

## 5. Conclusions

Insulin administration via IN alleviates autism-like behavioral, biochemical, and histopathological alterations induced by PPA in rats through modulation of signaling and anti-inflammatory pathways. Additionally, it facilitates pro-regeneration and repair via the GDF-15 pathway, highlighting the need for further studies to elucidate this mechanism.

## Figures and Tables

**Table 1:** Comparison of brain cytokines and protein levels in groups. Results are presented as mean ± SEM. Statistical analyses were performed by one-way ANOVA. * *p* < 0.01, ** *p* < 0.001 different from normal groups; # *p* < 0.05, ## *p* < 0.001 different from the PPA and saline group.

	Group 1 Normal Control	Group 2 PPA + Saline	Group 3 PPA + Insulin
Brain MDA level (nmol/gr protein)	50.1 ± 0.9	164.5 ± 9.5 **	97.1 ± 8.2 ##
Brain TNF-alfa level (pg/mg protein)	12.5 ± 1.1	196.1 ± 12.3 **	91.8 ± 7.7 ##
Brain IL-2 level (pg/mg protein)	2.1 ± 0.2	215.2 ± 14.1 **	85.1 ± 9.3 ##
Brain IL-17 level (pg/mg protein)	195.3 ± 20.1	524.5 ± 17.4 *	412.7 ± 10.6 #
Brain GDF-15 level (pg/mg protein)	13.08 ± 0.6	19.3 ± 0.8 **	24.05 ± 1.1 #
Brain NGF level (pg/mg protein)	78.3 ± 2.4	39.4 ± 3.1 **	66.3 ± 5.4 #

**Table 2:** Comparison of behavioral tests in groups. Results are presented as mean ± SEM. Statistical analyses were performed by one-way ANOVA., * *p* < 0.001 different from normal groups; # *p* < 0.05, ## *p* < 0.001 different from the PPA and saline group.

	Group 1 Normal Control	Group 2 PPA + Saline	Group 3 PPA + Insulin
Sociability test: The percentage (%) of time spent with a stranger rat	65.9 ± 3.5	29.4 ± 2.2 *	63.8 ± 3.5 ##
Open Field Test: Amount of ambulation	17.6 ± 2.5	8.3 ± 1.9 *	15.4 ± 2.1 #
Passive avoidance learning (PAL) Latency (Sec.)	266.4 ± 19.5	85.4 ± 20.5 *	214.5 ± 16.2 ##

**Table 3:** Comparison of neuronal counts and Glial Fibrillary Acidic Protein (GFAP) immunostaining indexes in groups. Results are presented as mean ± SEM. Statistical analyses were performed by one-way ANOVA. * *p* < 0.01, ** *p* < 0.001 different from normal groups; # *p* < 0.05, ## *p* < 0.001 different from the PPA and saline group.

	Group 1 Normal Control	Group 2 PPA + Saline	Group 3 PPA + Insulin
Neuronal Count CA1	81.2 ± 3.9	65.3 ± 4.8 **	77.9 ± 1.8 ##
Neuronal Count CA3	44.3 ± 3.7	34.5 ± 3.1 **	39.8 ± 6.3 #
GFAP immunostaining index (CA1)	35.8 ± 1.4	46.9 ± 4.06 *	41.7 ± 0.8 #
GFAP immunostaining index (CA3)	33.3 ± 0.9	42.1 ± 2.3 *	35.7 ± 1.5 #
Purkinje Count Cerebellum	21.7 ± 1.3	15.2 ± 0.8 *	18.6 ± 1.2 #
GFAP immunostaining index (Cerebellum)	21.4 ± 2.3	27.1 ± 2.9 *	22.5 ± 1.8 #

## Data Availability

All the data for this study are presented in the published article. Any further details are available from the corresponding author (Ejder Saylav Bora ejdersaylav.bora@ikc.edu.tr) upon a reasonable request.
